# Inhibiting the pathological changes of PASMCs is an effective approach for medicinal plants or secondary metabolites in treating pulmonary hypertension

**DOI:** 10.3389/fphar.2026.1725809

**Published:** 2026-02-12

**Authors:** Qi Liang, Hanghang Gou, Yi Zhu, Lin He, Liping Chen, Chuantao Zhang, Li Ai

**Affiliations:** 1 School of Pharmacy, School of Ethnic Medicine, Chengdu University of Traditional Chinese Medicine, Chengdu, China; 2 Hospital of Chengdu University of Traditional Chinese Medicine, Chengdu, China; 3 Sichuan Institute for Drug Control, Chengdu, China

**Keywords:** mechanism, medicinal plants or secondary metabolites, PASMCs, pathological changes, pH

## Abstract

Pulmonary hypertension (PH) is a progressive cardiovascular disease characterized by increased pulmonary vascular resistance and structural remodeling of pulmonary vessels, leading to poor clinical outcomes and high mortality. pulmonary artery smooth muscle cells (PASMCs) migration, apoptosis and abnormal proliferation are the main pathological features leading to the occurrence of PH. Increasing evidence suggests that inhibition of PASMCs pathological changes contributes to the improvement of PH. However, the current clinical treatment of PH is limited, and medicinal plants or secondary metabolites are gradually recognized as potential treatment options for PH. Therefore, this article focuses on inhibiting the abnormal pathological changes of PASMCs, and analyzes and summarizes the mechanism and process of medicinal plants or secondary metabolites in the treatment of PH by inhibiting the abnormal proliferation of PASMCs, so as to provide a direction for the development of medicinal plants or secondary metabolites for the treatment of PH.

## 1 Introduction

Pulmonary hypertension (PH) is a severe syndrome characterized by pulmonary vascular obstruction, persistently elevated pulmonary vascular resistance, right ventricular hypertrophy, and progressive functional decline (Zhang et al., 2022). Its core pathological mechanism is pulmonary artery remodeling, which is mainly characterized by dysfunction of pulmonary artery endothelial cells, excessive proliferation of pulmonary artery smooth muscle cells (PASMCs), activation of fibroblasts, and inflammatory cell infiltration. Among them, the imbalance between abnormal proliferation and apoptosis of PASMCs is particularly critical (Cuthbertson et al., 2023; Jiao et al., 2025; Lu et al., 2021).

At present, the global prevalence of PH is about 1% (Yang et al., 2025), and it shows an aging trend. Inhaled vasodilators and nitric oxide are commonly used in clinical practice to improve cardiac function and hemodynamics, but long-term use is prone to drug resistance, side effects and economic burden. Medicinal plants and their secondary metabolites have the characteristics of abundant sources, few adverse reactions, and low cost, which have shown potential in the treatment of PH (Ivy et al., 2024). In particular, some active ingredients can play a role by inhibiting PASMCs (Li M. X. et al., 2016; Miao et al., 2025; Qian et al., 2024). However, there is a lack of systematic analysis of the anti-pulmonary hypertension effects of medicinal plants or secondary metabolites by regulating PASMCs. Therefore, this review focuses on the latest research progress of the role and mechanism of PASMCs in PH, in order to provide new ideas for the development of future treatment strategies.

## 2 Normal functions of PASMCs

**FIGURE 1:**
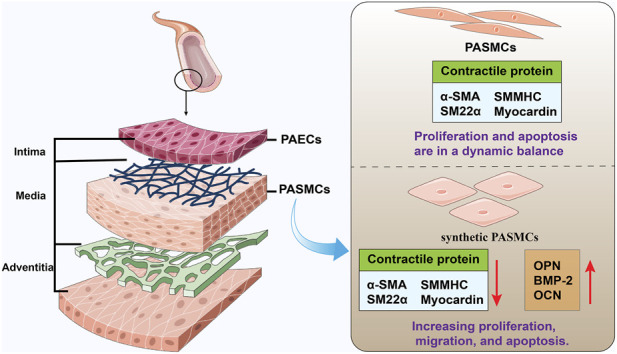
Distribution and function of PASMCs.

PASMCs are highly differentiated cells in blood vessels with contractile and diastolic functions. They regulate blood vessels by contraction and relaxation, thereby promoting blood circulation and regulating blood pressure and blood flow (Cowan et al., 2003). PASMCs are capable of transitioning between a differentiated, contractile phenotype and a dedifferentiated, synthetic phenotype in response to varying extracellular stimuli. Under normal physiological conditions, PASMCs maintain a relatively quiescent state following their differentiation and maturation into the contractile phenotype (Jin et al., 2018). PASMCs express smooth muscle α-actin (α-SMA), smooth muscle 22α (SM22α) and myocardin, all of which are essential for maintaining the contractile function of blood vessels. The expression of contractile protein in synthetic PASMCs gradually decreased or lost, while the expression of osteopontin (OPN) and bone morphogenetic protein 2 (BMP-2) increased (Allahverdian et al., 2018). In addition, differentiated contractile PASMCs are generally spindle-shaped or fusiform, with strong cell contraction ability, and proliferation and apoptosis are in a dynamic balance, which is mainly responsible for maintaining vascular wall tension and vascular elasticity (Angelini et al., 2013; Gao et al., 2018; Yu et al., 2019; Yue et al., 2019). The synthetic PASMCs were polygonal, with large cell volume and increased proliferation, migration, and apoptosis (Figure 1).

## 3 The effects and mechanisms of medicinal plants or secondary metabolites on PASMCs in PH

PVR is a key determinant in the progression of pulmonary hypertension. In the early stages of this remodeling process, blood vessels activate a compensatory mechanism aimed at maintaining normal physiological function (Guignabert and Dorfmuller, 2013). However, if vascular homeostasis is broken due to improper repair, excessive proliferation of PASMCs in the injured area will destroy the normal physiological morphology and function of the vessel wall. In general, PVR is a structural change caused by adaptive changes and repair of damaged vessels (Lacolley et al., 2018; Zhu et al., 2019). It has been reported that individuals residing at high altitudes frequently demonstrate right ventricular hypertrophy and increased pulmonary vascular pressure, primarily attributed to irreversible PVR resulting from prolonged exposure to hypoxia (Lee et al., 2019). Moreover, studies have found obvious vascular occlusion and vascular remodeling in PH mouse models. The pathological changes in PASMCs, including excessive proliferation, phenotypic transformation, migration, and apoptosis, are key contributors to PVR in PH.

Medicinal plants or secondary metabolites from natural sources, which are potential drugs for treating diseases. In recent years, substantial evidence has demonstrated that ameliorating PVR through the normalization of PASMCs represents an effective therapeutic strategy for PH. Furthermore, targeting the pathological alterations in PASMCs may offer a promising avenue for the development of novel PH treatments. In this section, we summarize the effects and possible mechanisms of medicinal plants or secondary metabolites on the proliferation, migration, and apoptosis of PASMCs in PH, all relevant medicinal plants or secondary metabolites are summarized in Table 1.

**TABLE 1:** Natural compounds targeting PASMCs dysfunction in PH.

Ways	Herbs/Secondary metabolites	Stucture	Biological activity	Related molecular targets	Effective dose	Model	Ref
Migration	Quercetin	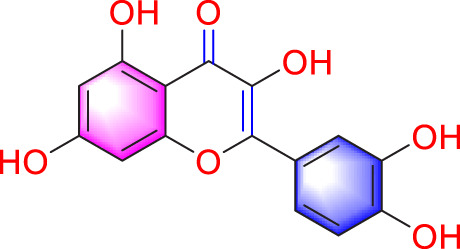	Inhibiting the migration and proliferation of PASMCs, and promoting their apoptosis	Inhibiting the TrkA/AKT signaling pathway	100 mg/kg/d	SD rats; PASMCs	He et al. (2015)
			Inhibiting the migration, proliferation and phenotypic transformation of PASMCs	Downregulating the TGF-β1/Smad2/Smad3 pathway	100 mg/kg/d	SD rats; PASMCs	Gao et al. (2024)
	Resveratrol	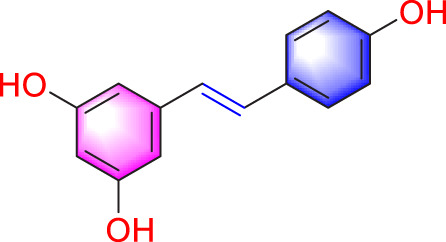	Inhibiting the migration and proliferation of PASMCs	Downregulating expression and phosphorylation of AKT	10 μmol/L	SD rats; PASMCs	Guan et al. (2017)
	Schisandrin B	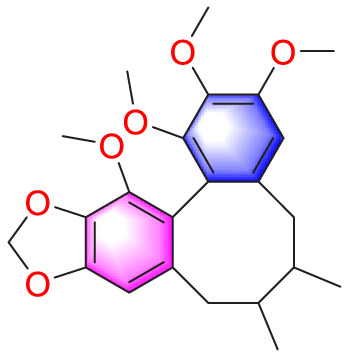	Inhibiting the migration and proliferation of PASMCs, and promoting their apoptosis	Inhibiting the TGF-β1 and Activating downstream signaling pathways	20, 50, 100, 150 μM	PASMCs	Wu et al. (2017a)
​	Berberine	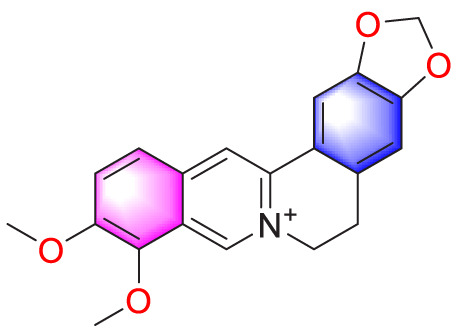	Inhibiting the migration and proliferation of PASMCs	Regulating the PP2A signaling pathway	100 mg/kg	PASMCs	Luo et al. (2018)
			Inhibiting the migration and proliferation of PASMCs	Inhibiting the Src phosphorylation and suppressing the HIF-1α expression through Akt/mTOR signaling pathway	100 mg/kg	C57/BL6 mice; PASMCs	Liu et al. (2019)
	Andrographolide	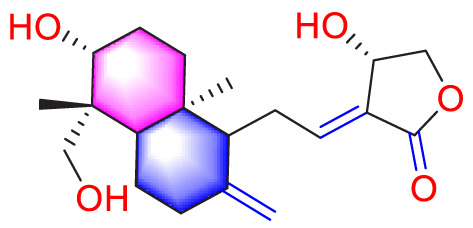	Inhibiting the migration and proliferation of PASMCs, and promoting their apoptosis	Inhibiting the TLR4/NF-κB, ERK, and JNK-MAPK signaling pathways Activating the p38-MAPK	1 mg/kg/d	C57BL/6J mice; PASMCs	Nie et al. (2021)
	Parthenolide	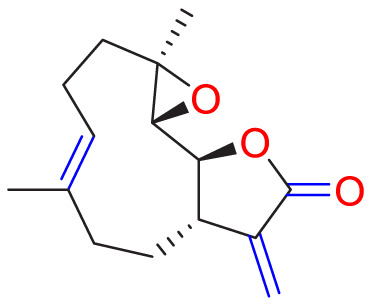	Inhibiting the migration and proliferation of PASMCs	Suppressing STAT3 activity	30 mg/kg	SD rats; PASMCs	Yao et al. (2024)
	Dehydrodiisoeugenol	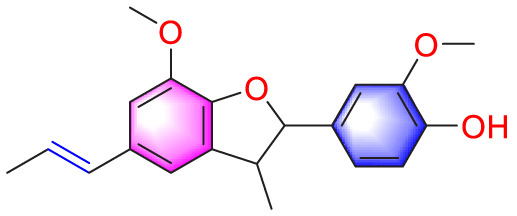	Inhibiting the migration and proliferation of PASMCs, and promoting their apoptosis	Suppressing the mTOR/HIF1-α/HK2 signaling pathway	20 µM	PASMCs	Xie et al. (2025)
Apoptosis	Puerarin	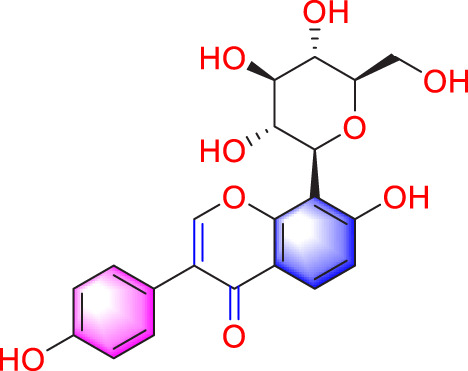	Promoting apoptosis of PASMCs	Activating the caspase-9, downregulating Bcl-2, upregulating of Bax	50 µM	PASMCs	Chen et al. (2012)
	Plumbagin	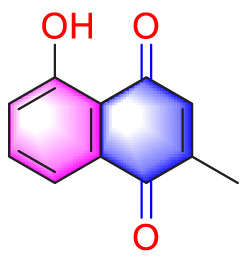	Inhibiting the proliferation of PASMCs and promoting apoptosis	Inhibiting the STAT3/NFAT axis activation	4 mg/kg	SD rats; PASMCs	Courboulin et al. (2012)
	Salidroside	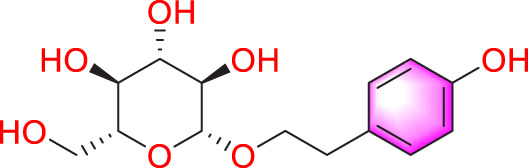	Promoting apoptosis of PASMCs	Upregulating the expression of A2aR	0, 16, 32, and 64 mg/kg	BALB/C mice; PASMCs	Huang et al. (2015)
			Inhibiting the proliferation of PASMCs and promoting apoptosis	Activating the AMPKα1-P53-P27/P21 pathway	8 mg/kg	SD rats; PASMCs	Chen et al. (2016)
	Carvacrol	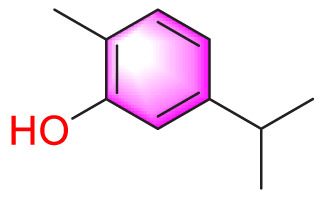	Promoting apoptosis of PASMCs	Inhibiting the ERK1/2 and PI3K/Akt pathway, Bcl-2 expression, and promoting caspase-3 activation	50 mg/kg	Wistar rats; PASMCs	Zhang et al. (2016)
​	Baicalin	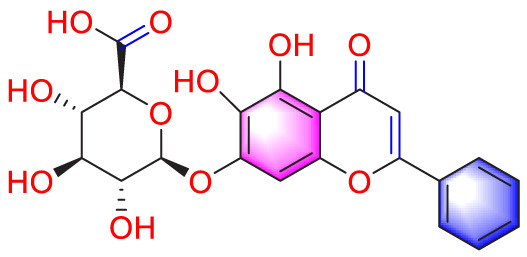	Inhibiting the proliferation of PASMCs and promoting apoptosis	Inhibiting the NF-κB signaling Activating the BMP signaling	100 mg/kg	Wistar rats; PASMCs	Zhang et al. (2017a)
	Aloperine	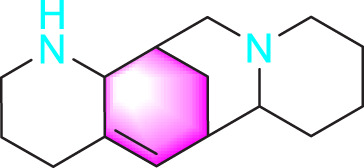	Promoting apoptosis of PASMCs	Inhibiting the Rho A and ROCK Downregulating of Bcl-2/Bax ratio	50 mg/kg	SD rats	Wu et al. (2017b)
			Inhibiting the proliferation of PASMCs and promoting apoptosis	Inhibiting the NK-κ B signaling pathway and upregulating p27	0.125, 0.25, 0.5, 1 mM	PASMCs	Chang et al. (2019)
	Emetine	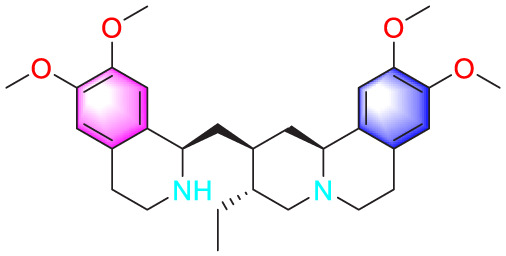	Inhibiting the proliferation of PASMCs and promoting apoptosis	Downregulating the expressions of RhoA/Rho-kinases Reducing the secretion of CyPA, BSG	0.05 mg/kg/d	SD rats; PASMCs	Siddique et al. (2019)
	Astragaloside IV	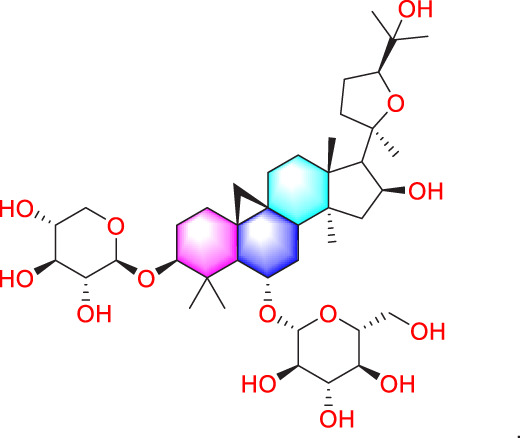	Inhibiting the proliferation of PASMCs and promoting apoptosis	Inhibiting the HIF-1 α and p-ERK1/2 protein expression, upregulating of Bax, cleaved caspase-9, and cleaved caspase-3 levels	20 μM	PASMCs	Jin et al. (2021)
​	Pachymic acid	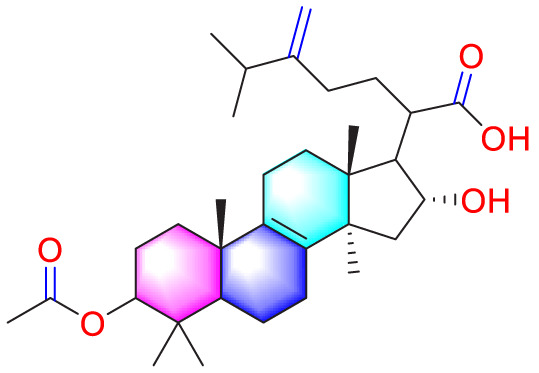	Inhibiting the proliferation of PASMCs and promoting apoptosis	Activating the Nrf2-Keap1-ARE signaling pathway	5 mg/kg/d	SD rats; PASMCs	He et al. (2022)
	Juglone	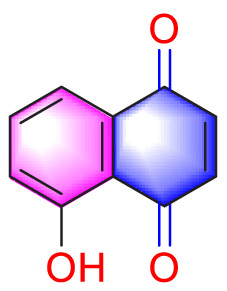	Inhibiting the proliferation of PASMCs and promoting apoptosis	Inhibiting Pin1	1.5 mg/kg	Rats; PASMCs	Rai et al. (2022)
	Kaempferol	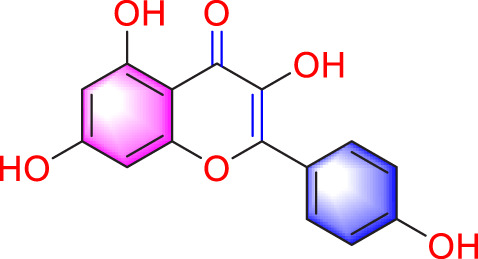	Inhibiting the proliferation of PASMCs and promoting apoptosis	Regulating the Akt/GSK3β/CyclinD axis	25 mg/kg/d	SD rats; PASMCs	Zhang et al. (2023)
	Osthole	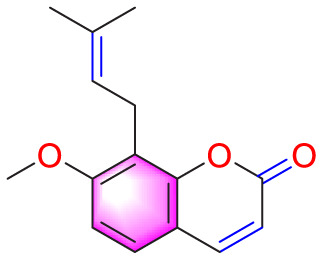	Promoting apoptosis of PASMCs	Upregulating the ASK1 and Bax/Bcl-2-Aspase3 signaling pathways	20 mg/kg	SD rats; PASMCs	Li et al. (2017), Zhu et al. (2021b)
​	Luteolin	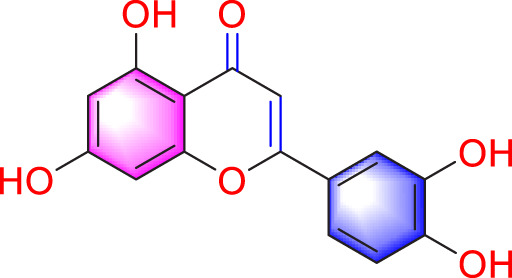	Inhibiting the proliferation of PASMCs and promoting apoptosis	Upregulating Kv1.5	50 μM	SD rats; PASMCs	Çetinkaya and Baran (2023), Ji et al. (2022), Zhang et al. (2024)
Proliferation	1,8-Cineole	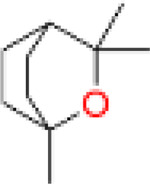	Inhibiting PASMCs proliferation	Activating BMPR2 signaling pathway	25 mg/kg or 100 mg/kg	Wistar rats	Alves-Silva et al. (2025)
	Celastramycin	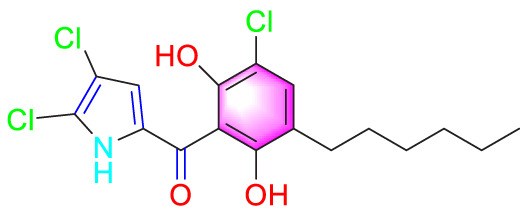	Inhibiting excessive proliferation of PASMCs	Inhibiting the HIF-1 α and NF - κ B levels, and reducing the cytokines/chemokines	3 mg/kg/d	Rats	Kurosawa et al. (2019)
	Ginsenoside CK	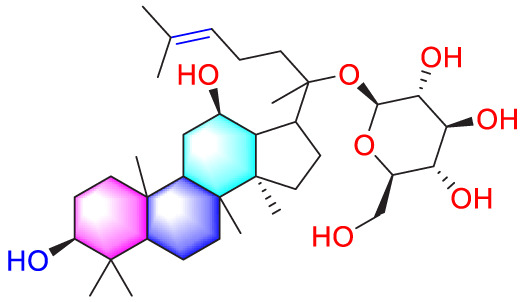	Inhibiting PASMCs proliferation and reversing the phenotypic transformation of PASMCs	Inhibiting the WNT/β-catenin signaling pathway	5 μmol/L	PASMCs	Liu et al. (2021)
	Cannabidiol	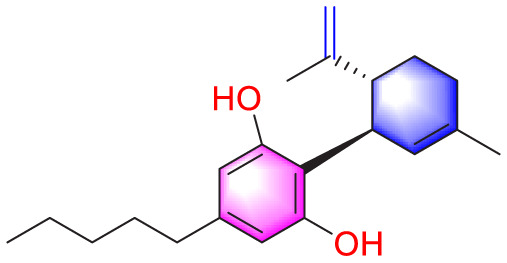	Inhibiting PASMCs proliferation	Activating Nrf2 and its downstream proteins	10 mg/kg/d	C57BL/6J mice; SD rats	Lu et al. (2021), Sadowska et al. (2020)
​	Crocin	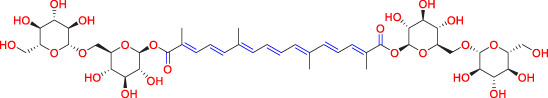	Inhibiting PASMCs proliferation	Inhibiting the CCL2/CCR2 pathway	—	Rats; PASMCs	Sheng et al. (2022)
	Tanshinone IIA	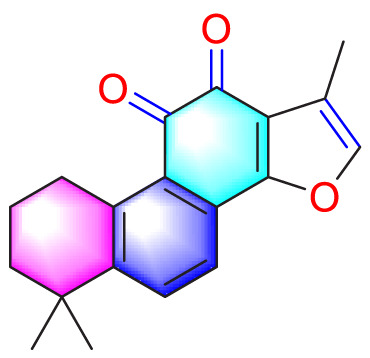	Inhibiting PASMCs proliferation	Inhibiting the Akt/Skp2 related pathways	30 μg/mL	PASMCs	Luo et al. (2013)
	Danshensu	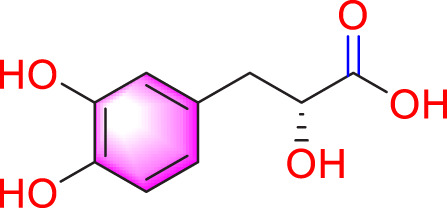	Inhibiting PASMCs proliferation	Inhibiting the TGF-β-Smad3 related pathway	160 mg/kg/d	SD rats; PASMCs	Zhang et al. (2018b)
	Epigallocatechin-3-gallate	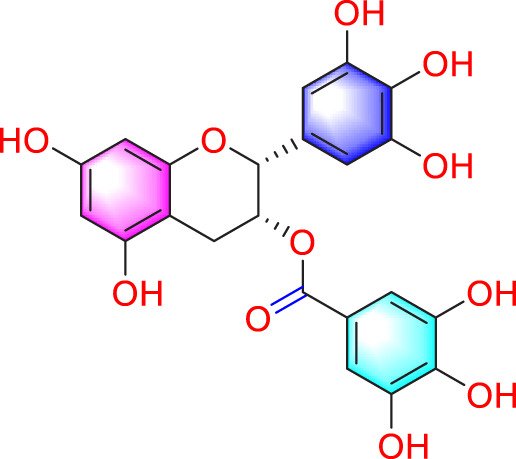	Inhibiting PASMCs proliferation	Upregulating the KLF-4 and MFN-2, downregulating the p-Erk	50 mg/kg/d	SD rats; PASMCs	Zhu et al. (2017)
​	Isorhynchophylline	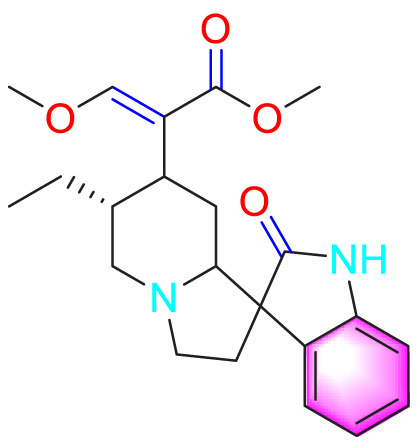	Inhibiting PASMCs proliferation	Reducing cyclin D1, ERK1/2, p-STAT3, Akt/GSK3 β Increasing the accumulation of p27Kip1	1000 mg/kg	Rats; PASMCs	Guo et al. (2014)
	Halofuginone	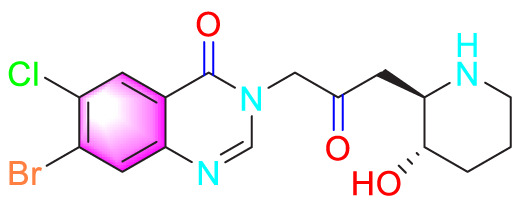	Inhibiting PASMCs proliferation	Inhibiting the PI3K/Akt/mTOR signaling pathway	0.15 mg/kg/d	C57B1/6 mice; PASMCs	Jain et al. (2021)
	Ligustrazine	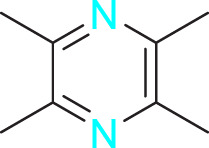	Inhibiting PASMCs proliferation	Reducing the phosphorylation expression of PI3K and AKT	160 mg/kg/d	Rats; PASMCs	Huang et al. (2021)
	Paeoniflorin	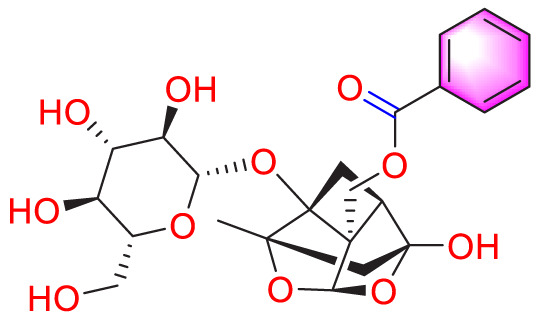	Inhibiting PASMCs proliferation	Activating A2BAR	20 μmol/L	PASMCs	Qian et al. (2013)
	Magnolol	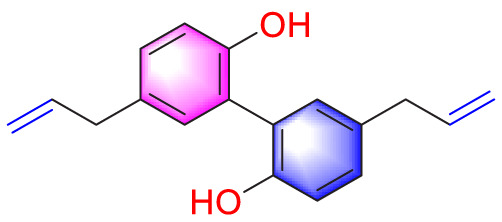	Inhibiting PASMCs proliferation and phenotypic transformation	Inhibiting the JAK2/STAT3 pathway	10 mg/kg	SD rats; PASMCs	Xiao et al. (2022)
​	Hydroxysafflor yellow A	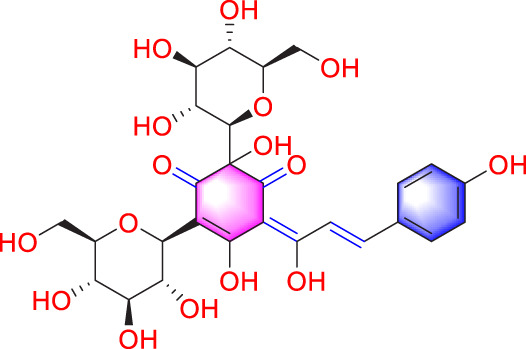	Inhibiting PASMCs proliferation	Inhibiting the protein expression of PCNA	25 mg/kg	Wistar rats; PASMCs	Li et al. (2016b)
	Tetrandrine	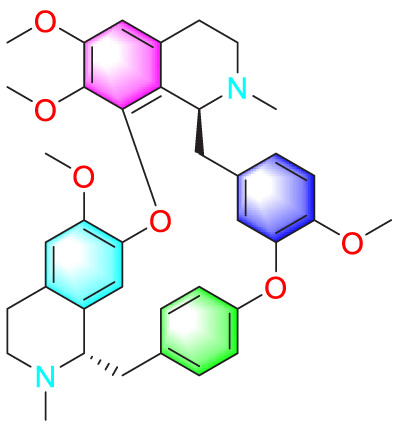	Inhibiting PASMCs proliferation	Upregulating the PKG-1 and downregulating the iNOS	50 mg/kg	SD rats	Wang et al. (2016)
	Oxymatrine	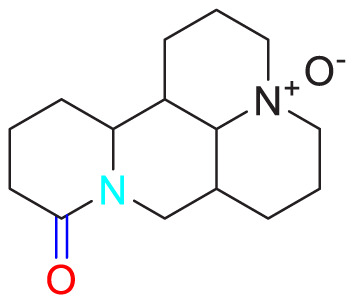	Inhibiting PASMCs proliferation	Upregulating the Nrf2 and antioxidant protein expression	50 mg/kg/d	SD rats; PASMCs	Zhang et al. (2014)
	Berberine	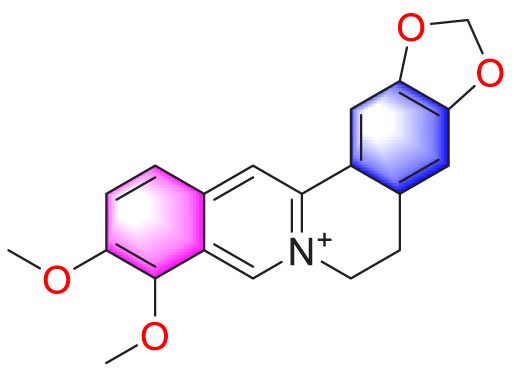	Inhibiting PASMCs proliferation	Inhibiting the Trx1 and its target gene β-catenin expression	10 μmol/L	PASMCs	Wande et al. (2020)
​	Osthole	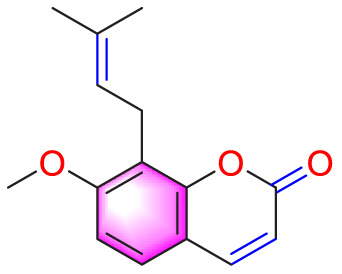	Inhibiting PASMCs proliferation	Inhibiting the TGF-β 1/Smad/p38 signaling pathway	10 μM	PASMCs	Yue et al. (2020)
			Inhibiting PASMCs proliferation	Downregulating miRNA-22-3p levels and decreasing the accumulation of PCNA, FAS, CPT1A, and HK2	80 mg/kg; 100 nM	SD rats; PASMCs	Niu et al. (2022)
	Isoquercitrin	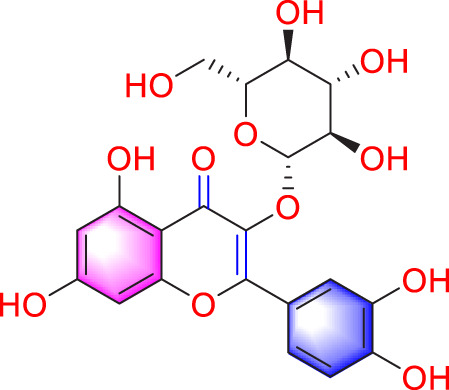	Inhibiting PASMCs proliferation	Inhibiting the PDGF-R β signaling pathway	30 μmol/L	Wistar rats; PASMCs	Zhang et al. (2017b)
	Puerarin	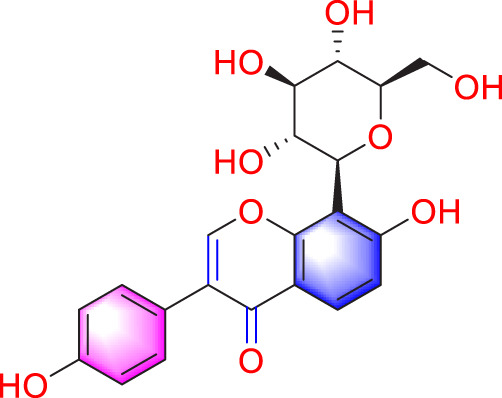	Inhibiting PASMCs proliferation	Inhibiting the PCNA expression and autophagy activation	80 mg/kg/d	Wistar rats; PASMCs	Zhang et al. (2019)
	Safflower injection	​	Inhibiting PASMCs proliferation	Reducing the ratio of TXB2/6-keto-PGF1α	2 mL/kg	SD rats	Zeng et al. (2009)
	Yarsagumba extract	​	Inhibiting PASMCs proliferation	—	0.50 mM	C57BL/6 mice; PASMCs	Luitel et al. (2020)

### 3.1 Migration of PASMCs

**FIGURE 2:**
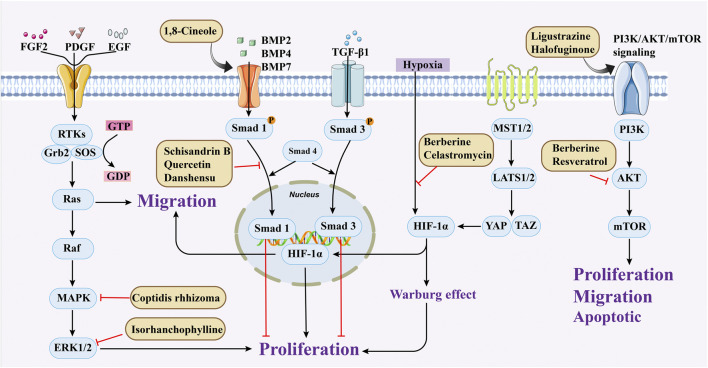
Medicinal plants or secondary metabolites regulate PASMCs cell proliferation and migration through different pathways.

Cell migration refers to the directed movement of cells, which is driven by changes in cell morphology in response to external signaling stimuli (Tajsic and Morrell, 2011; Yao et al., 2024). The migration of PASMCs plays a critical role in the development of neointimal formation, neovascularization, and filamentous lesions. The available evidence suggests that PASMCs migration is also regulated by PDGF and can stimulate PASMCs migration from the media to the neointima, and that neointimal thickening is significantly reduced when migration is inhibited by antiplatelet or anti-PDGF antibodies, whereas treatment with PDGF after vascular injury results in marked neointimal thickening (Sun et al., 2025). In addition, matrix metalloproteinase (MMP), and renin-angiotensin system (RAS) were identified to influence PASMCs migration (Jiao et al., 2023). Increased expression of epidermal growth factor (EGF), fibroblast growth factor 2 (FGF2), and extracellular matrix secondary metabolites collagen, fibronectin, laminin, and tenascin was found (Zhang Y. X. et al., 2018). It also has the effect of promoting cell migration, thereby participating in PVR in PH (Zeng et al., 2019). (Figure 2).

*Coptis chinensis* Franch., a plant in the Ranunculaceae, contains multiple bioactive compounds. Through the establishment of a rat pulmonary arterial hypertension treatment model, the empirical research results of Luo et al. showed that the effective ingredients of *C. chinensis* Franch can inhibit the expression of MAPK1, effectively inhibit the migration and proliferation of PASMCs, thereby alleviating pulmonary artery remodeling (Luo et al., 2021). In addition to medicinal plants or secondary metabolites, the importance of traditional Chinese medicine formulas cannot be ignored, and compound preparations are relatively commonly used in clinical treatment. In a study conducted in 2021, it was recorded that Xinmai’an extract (containing *Panax ginseng* C.A.Mey., *Astragalus membranaceus* Fisch. ex Bunge., *Salvia miltiorrhiza* Bunge, *Paeonia lactiflora* Pall., *Ophiopogon japonicus* (Thunb.) Ker Gawl., and *Dryobalanops aromatica* C.F.Gaertn.) can inhibit the MAPK signaling pathway and increase MMP2 and MMP9, effectively alleviating the migration, proliferation, and anti-apoptosis of PASMCs (Zhu Y. et al., 2021).

In 2015, He et al. found through *in vitro* experiments that quercetin (Que, 10, 30, and 60 μmol/L) may dose dependently reduce the migration of PASMCs by inhibiting the TrkA/AKT signaling pathway (He et al., 2015). In addition, a recent study has shown that Que has more than one anti migration pathway. Through *in vitro* (60 μmol/mL) and *in vivo* (5 mg/kg/d) studies, it has been demonstrated that Que suppress PVR by inhibiting the TGF-β1/Smad2/Smad3, reducing migration, proliferation, and phenotype transformation of PASMCs (Gao et al., 2024).

Resveratrol is a phenolic compound primarily obtained from the Polygonaceae plant *Reynoutria japonica* Houtt. In 2017, Guan et al. established a hypoxia model using rat PASMCs primary cells and found that resveratrol (10 μmol/L) could inhibit phosphorylation of AKT, thereby reducing the migration and proliferation of PASMCs (Guan et al., 2017). Schisandrin B (Sch B) is one of the major bioactive constituents found in *Schisandra chinensis* (Turcz.) Baill. Research has shown that at any doses (20, 50, 100, 150 μM), Sch B has a strong therapeutic effect by reducing TGF-β1 levels and activating downstream signaling pathways, thereby alleviating PASMCs migration and apoptosis resistance caused by hypoxia (Wu J. et al., 2017). Berberine (BBR) occurs naturally in various plant species and has been extensively utilized in the treatment of conditions such as gastroenteritis and bacterial dysentery. In 2018, Luo et al. found that BBR (100 mg/kg) may suppress migration and proliferation of PASMCs in PH models induced by norepinephrine by activating the PP2A (Luo et al., 2018). Subsequent research in 2019 found that BBR, as a Src inhibitor, can inhibit Src activation and HIF-1α expression, thereby suppressing Akt/mTOR and slowing down the migration and proliferation of PASMCs (Liu et al., 2019).

Andrographolide (ANDR) derived from the medicinal plant *Andrographis paniculata* (Burm.f.) Wall. ex Nees. It exhibits a broad spectrum of anti-cancer activities and demonstrates significant therapeutic potential in the context of cardiovascular diseases (Gou et al., 2023; Hu et al., 2024). Due to its strong anti-proliferative activity in the treatment of cancer, ANDR has attracted researchers to explore its potential therapeutic role in the process of PVR towards PH. Nie et al. systematically studied its related mechanisms through *in vitro* and *in vivo* experiments. ANDR (1 mg/kg/day) restored the signal transduction of BMPR2, inhibited the activation of TLR4/NF-κB and NOX/Nrf2, and jointly caused a reduction in the proliferation and migration of PASMCs, and promoted their apoptosis, demonstrating a multi-target effect (Nie et al., 2021). Taken together, these findings highlight the therapeutic potential of medicinal plants or secondary metabolites for PH by inhibiting multiple molecular mechanisms such as TGF-β1/Smad2/Smad3 axis, TLR4/NF-κB and NOX/Nrf2 pathways to inhibit the migration of PASMCs and improve the pathological symptoms of PASMCs.

### 3.2 Apoptosis of PASMCs

**FIGURE 3:**
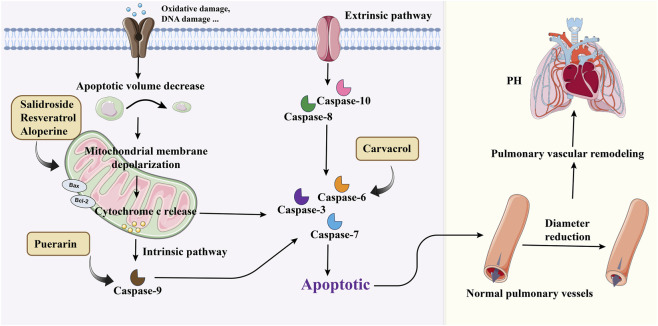
Medicinal plants or secondary metabolites regulate PASMCs apoptosis through different pathways. (Extrinsic pathway for caspase 8 and 10 and intrinsic pathway for caspase 9. Activation of different starting caspases eventually leads to activation of the same effector caspases 3, 6, and 7).

Apoptosis, a known programmed cell death pathway, is an important way for multicellular organisms to maintain homeostasis in the internal environment (Liu et al., 2025). Apoptosis normally removes cells that migrate into the vascular lumen and eliminates accumulated mast cells within the pulmonary vasculature. A reduction in apoptosis, coupled with increased PASMCs proliferation, contributes to vascular wall thickening, and elevated pulmonary vascular resistance, ultimately leading to the development of PH (Perini et al., 2018). It has been reported that inhibitors of Bcl-2 and Bcl-xl in the Bcl family of anti-apoptotic proteins can promote apoptosis and thus delay the development of PH (Rybka et al., 2018). Therefore, promoting apoptosis can reverse PVR and is a potential therapeutic option against PH (Figure 3).

Puerarin (Pue) is an isoflavone derivative isolated from *Pueraria lobata* (Willd.) Ohwi. It is often used in diseases such as heart disease and high blood pressure. As early as 2008, some scholars found that Pue could treat PVR in PH rats, but the specific therapeutic mechanism was not mentioned (Li et al., 2008). Subsequently, Chen et al. conducted further studies by culturing HPASMCs *in vitro* and found that Pue treatment with 50 μM and above could upregulate caspase-9 and Bax, downregulate Bcl-2, and promote cell apoptosis, thereby determining the possible related mechanism (Chen et al., 2012). Salidroside (SAL), a diterpenoid compound from the rhizome of *Rhodiola rosea* L. (Kosanovic et al., 2013), treated chronic hypoxia mice with different concentrations of SAL (0, 16, 32 and 64 mg/kg). It was found that SAL could activate AMPKα1-P53-P27/P21 to reduce PASMCs proliferation, thereby attenuating chronic hypoxia-induced PH (Huang et al., 2015). Moreover, it promotes cell apoptosis through Bax/Bcl-2-caspase 9-caspase 3 to alleviate pulmonary artery remodeling, thereby alleviating PH (Chen et al., 2016). Interestingly, Aloperine (Alo) is a quinoline alkaloid mainly extracted from the seeds and leaves of *Sophora alopecuroides* L., and it possesses various biological activities, including anti-inflammatory, anticancer. Researchers found that Alo (10 mg/kg) could downregulate Bcl-2/Bax ratio, promote cell apoptosis, and improve PH-related symptoms in rats (Wei et al., 2025). Subsequently, Chang et al. further explored the mechanism of Alo treatment by using HPASMCs to supplement Alo for PH, and found that Alo (0.125, 0.25, 0.5, 1 mM) could enhance cell apoptosis by inhibiting NK-κB and increasing p27. Thus, the excessive proliferation of PASMCs was reduced (Chang et al., 2019).

Plumbagin (PLU) is a naphthoquinone substance mainly found in the herbaceous plant *Plumbago zeylanica* L., which has anti-tumor and anti-proliferative pharmacological effects. It is often used as a STAT3 inhibitor in cancer cells to promote cell apoptosis (Hsu et al., 2006). Its pro apoptotic properties are also highly valued in pulmonary arterial hypertension. Researchers have identified a strong correlation between the proliferation and anti-apoptotic effects on PASMCs and the activation of STAT and NFAT signaling pathways, and PLU has been shown to specifically target STAT. A thorough study by Courboulin et al. showed that *in vivo*, Oral PLU (4 mg/kg) can reduce the distal pulmonary artery remodelling, mean pulmonary artery pressure and right ventricular hypertrophy without affecting systemic circulation in both monocrotaline-and suden/chronic hypoxia-induced PH in rats can inhibit the activation of STAT3/NFAT axis and alleviate the apoptosis resistance and excessive proliferation of PASMCs (Courboulin et al., 2012).

Although traditional Chinese medicine secondary metabolites have significant pharmacological effects, their effects may have many targets, such as affecting different pathways to achieve the same results. Quercetin is a typical example. In addition to balancing PASMCs by inhibiting the TrkA/AKT and the TGF-β1/Smad2/Smad3, other researches have shown that it can also work by upregulating 5-HT2A receptors, restoring Kv current, and reducing phosphorylation of AKT and S6. These effects guide the apoptosis of PASMCs and inhibit its proliferation, thereby reducing the mortality rate of PVR rats (Morales-Cano et al., 2014). Carvacrol (CAR) is a monoterpene phenol and one of the main secondary metabolites of beef tallow (Burt, 2004). In tumor cells, it also exhibits pro apoptotic properties. Scholars hypothesize that it can also be applied to pulmonary arterial hypertension and have found that CAR can induce apoptosis of PASMCs by inhibiting the ERK1/2 and PI3K/Akt, reducing Bcl-2 expression, and promoting caspase-3 activation, providing new insights for the treatment of hypoxic PH (Zhang et al., 2016). Baicalin, a flavonoid and main bioactive compound, is found in *Scutellaria baicalensis* Georgi, a commonly used herb in traditional Chinese medicine. It can be used to treat cancer, liver and intestinal diseases, and acute lung injury (He et al., 2021; Hu et al., 2021; Li-Weber, 2009). In 2010, a survey in Cell Research found that baicalin inhibited the proliferation of PASMCs by inhibiting PDGFR β-ERK1/2 and the accumulation of p27, thereby reducing the development of atherosclerosis (Dong et al., 2010). The related phenotypes caused by it are highly similar to PASMCs in PH. Therefore, in the in-depth study by Zhang et al., it was found that baicalin can also improve PH induced by MCT in rats, by inhibiting NF-κB and activating bone morphogenetic protein (BMP) mechanisms to promote apoptosis and anti-proliferation of PASMCs (Zhang Z. et al., 2017).

As previously mentioned, resveratrol has been shown to inhibit the migration of PASMCs by downregulating the phosphorylation of AKT. In addition, the role of Res in cell apoptosis cannot be ignored. After establishing a rat model of PH, Yu et al. found that Res administration could improve PVR and right ventricular hypertrophy. *In vitro* cellular mechanism experiments have shown that Res can enhance the activation of SIRT1, induce mitochondrial permeability transition (mPT) dysfunction, enhance cell apoptosis, and thus resist the proliferation of PASMCs (Yu et al., 2017).

### 3.3 Proliferation of PASMCs

Cell proliferation and differentiation play a key role in tissue development. Excessive PASMCs proliferation can cause thickening of the pulmonary arteriole walls and narrowing of the lumen, leading to PVR (Solinc et al., 2022; Zucker et al., 2019). PDGF-BB, a member of the PDGF family, is a major regulator of PVR. It accelerates smooth muscle cell proliferation by up-regulating low-density lipoprotein receptor-related protein 1 (LRP1), leading to thickening of the pulmonary vascular media and promoting PH (Qin et al., 2022). This phenomenon may be attributed to the excessive production of ROS during PDGF-BB-induced PASMCs proliferation, which leads to the activation of ataxia-telangiectasia mutated protein (ATM) (Deng et al., 2022; Wujak et al., 2021), thereby inhibiting PASMCs proliferation.

In addition, hypoxia-inducible factor (HIF)-1α plays a key role in the development of pulmonary hypertension by regulating downstream genes that promote PASMCs proliferation. Other studies have shown that the energy metabolism of mitochondria in PH patients does not favor aerobic respiration, but is more inclined to supply energy through anaerobic respiration, which is called the “Warburg effect,” which leads to the excessive proliferation of PASMCs, promotes the thickening of the media, and then leads to the occurrence of PH (Arai et al., 2021). When PASMCs are stimulated by the above factors, BDNF-TrkB-ERK1/2 (65), BMP/TGF-β (Wei et al., 2023), PDGF/Ca^2+^ (Lan et al., 2024), PI3K-AKT-Mtor (Meng et al., 2019) signaling pathways are activated, which further affect cell proliferation and promote PVR in PH. Therefore, the inhibition of excessive proliferation of PASMCs can be considered a potential therapeutic strategy for patients with PH. (Figure 2).

1,8-Cineole is mainly derived from plants such as *Eucalyptus* L'Hér. and *Mentha canadensis* L., and is a monoterpene compound used as a natural aromatic oil, and spice (Ćavar Zeljković et al., 2021). In addition to the above purposes, 1,8-Cineole can also be used in the medical industry. The latest research shows that 1,8-Cineole (25 and 100 mg/kg) mitigated PAH-associated derailment of both BMPR2/Smad1/5 and BMPR2/PPAR-γ pathways and concomitantly reduced interstitial fibrosis and the arterial medial layer thickness in pulmonary arteries. (Alves-Silva et al., 2025). Ginsenoside compound K (GCK) is one of *P. ginseng* C.A.Mey. secondary metabolites, which has shown anti proliferative effects in cancer, and the proliferation of PASMCs has similar characteristics to cancer cells (Attele et al., 1999). So under the research of Liu et al., after intervention with 5 μmol/L GCK in a cell model, the drug downregulated β-catenin and cyclin, inhibited cell cycle circulation, thus reduced abnormal proliferation of PASMCs (Liu et al., 2023). In the same year, it was also found that cannabidiol has an inhibitory effect on the proliferation of PASMCs at 10 μM, with almost no cytotoxicity (Sadowska et al., 2020). Its possible mechanism is that it upregulates Nrf2 and downstream proteins, improving oxidative stress and mitochondrial function. Similarly, studies have shown that oxymatrine inhibits abnormal PASMCs proliferation and oxidative stress by enhancing Nrf2 and antioxidant protein expression, thereby improving PH (Liu et al., 2014; Zhang et al., 2014).

Crocin is a water-soluble carotenoid compound that can be isolated from *Crocus sativus* L. or *Gardenia jasminoides* J.Ellis (Deng et al., 2024). Another study found that crocin not only inhibits collagen fiber proliferation, but also reduces the proliferation of PASMCs by suppressing the CCL2/CCR2 inflammatory pathway, improving PVR induced by MCT in rats with PH (Sheng et al., 2022). The activity of the typical lipophilic component Tanshinone IIA is already very broad (Pang et al., 2016). In PH disease, it can alleviate pulmonary artery remodeling in diseased SD rats, mainly by downregulating Akt/Skp2/P27 pathway proteins and inhibiting PASMCs proliferation (Luo et al., 2013). In addition, another water-soluble component of *S. miltiorrhiza* Bunge, Danshensu, can inhibit the conduction of TGF-β-Smad3, thereby exerting anti proliferative effects (Zhang N. et al., 2018). From various research results, *S. miltiorrhiza* Bunge is a key traditional Chinese medicine for treating PH, which can add a new therapeutic drug for this type of disease.

Epigallocatechin-3-gallate present in green tea can inhibit proliferation by upregulating KLF-4 and MFN-2 and downregulating p-Erk (Zhu et al., 2017). Isorhanchophylline, a compound derived from *Uncaria rhynchophylla* (Miq.)Miq. ex Havil., can also downregulate ERK1/2 and cyclin D1, p-STAT3, Akt/GSK3β, and increase the accumulation of p27Kip1, jointly exerting an anti PASMCs proliferation effect (Guo et al., 2014). It is also worth noting that the PI3K/AKT plays a role in controlling the proliferation of PASMCs. Two studies have demonstrated that Ligustrazine and Halofuginone can inhibit this pathway, prevent cell cycle progression, and improve PH (Huang et al., 2021; Jain et al., 2021).

The diversity of natural products corresponds to a variety of anti-proliferative mechanisms, but they can achieve the same effect. In addition to several common pathways, researchers have also found that paeoniflorin may inhibit the proliferation of PASMCs by activating A2BAR, thereby blocking the cell cycle progression (Qian et al., 2013). Magnolol can inhibit the phosphorylation level of JAK2/STAT3, exert anti proliferative effects, and prevent pulmonary artery remodeling (Xiao et al., 2022). Hydroxysafflor yellow A can also inhibit proliferation and reverse vascular remodeling by reducing PCNA and Ki67 levels, and possibly synergistically activating Kv channels (Li L. et al., 2016). Tetrandrine derived from the traditional Chinese medicine *Stephania tetrandra* S.Moore can upregulate the expression of PKG-1 while inhibiting iNOS, balancing the NO signaling pathway, and exerting anti proliferative and antioxidant effects, alleviating PH symptoms in rats (Wang et al., 2016).

Most of the research on the treatment of PH with natural products focuses on exploring the effects of herbs and their secondary metabolites, while there is relatively less research on other drugs, such as animals and microorganisms. Interestingly, Kurosawa et al.'s study found that a benzoylpyrrole type compound found in bacteria, Celastromycin (Cel), can improve PH related symptoms. The specific mechanism involved is that Cel downregulates HIF-1α and NF-κB, restores mitochondrial function, and inhibits abnormal proliferation of PASMCs (Kurosawa et al., 2019). This study further enriches the variety of natural products for treating PH and expands new ideas for future drug development. In summary, existing studies fully reveal the potential of medicinal plants or secondary metabolites to treat pulmonary hypertension through multi-target intervention of PASMCs migration, apoptosis and proliferation. However, there is a deeper logic behind this: as a multi-pathway disorder, the pathological network of pulmonary hypertension has a significant “robustness,” that is, the inhibition of a single target is often offset by a compensatory pathway. The multi-target nature of natural products can systematically perturb this pathological network and break its stable state (He et al., 2025). For example, simultaneous inhibition of TGF-β/Smad and MAPK pathways may result in synergistic effects rather than simply additive. Future research should go beyond the linear model of “one component, one pathway” and apply multi-omics and systems biology approaches to quantify the overall remodeling effect of medicinal plants or secondary metabolites on PASMC signaling network. It also focuses on its ability to drive cell “state transitions” such as the transition from a proliferative state to an apoptotic state. This shift in thinking from “target inhibition” to “network remodeling” may provide a new paradigm for the development of more effective multi-target synergistic therapies.

## 4 Conclusion

The pathogenesis of PH is complex and involves the joint action of many factors, which leads to abnormal migration, apoptosis and excessive proliferation of PASMCs, and then leads to PVR, increased vascular resistance and increased vascular pressure. At present, there is still a lack of completely effective treatment in clinical practice. Medicinal plants or secondary metabolites have attracted more and more attention due to their high safety and abundant resource sources (Bharate and Lindsley, 2024; Peng et al., 2024). This review systematically summarizes the latest research progress of medicinal plants and their secondary metabolites to improve pulmonary arterial hypertension (PH) by regulating the pathological changes of PASMCs, such as inhibiting abnormal proliferation and migration and promoting apoptosis. Medicinal plants and their secondary metabolites alleviate PH mainly by directly interfering with PASMCs function or indirectly exerting anti-inflammatory and anti-oxidation effects, which provides an important basis for the development of new therapeutic candidates.

However, many challenges remain in translating relevant findings into clinical application. At present, most studies are limited to cell and animal models, and the disease process is different from that of humans, and the clinical research is limited, which may lead to bias in the evaluation of efficacy. In addition, systematic evaluation of the safety, toxicity, and pharmacokinetic properties (e.g., solubility, oral bioavailability) of candidate ingredients is still inadequate.Therefore, more well-designed clinical trials are needed in the future to scientifically verify its efficacy and safety. At the same time, more efforts should be focused on optimizing the drug administration strategy and dosage form design to improve patient compliance, and ultimately promote more potential natural products from experimental research to clinical translation.
